# The influence of computer-assisted surgery experience on the accuracy and precision of the postoperative mechanical axis during computer-assisted lateral closing-wedge high tibial osteotomy

**DOI:** 10.1186/s43019-019-0023-1

**Published:** 2019-12-18

**Authors:** Hyun Woo Lee, Sang Jun Song, Dae Kyung Bae, Cheol Hee Park

**Affiliations:** 1 Department of Orthopaedic Surgery, College of Medicine, Kyung Hee University, Seoul, Korea; 2 Department of Orthopaedic Surgery, Seoul Sacred Heard General Hospital, Seoul, Korea; 3 Department of Medicine, Graduate School, Kyung Hee University, 23 Kyunghee-daero, Dongdaemun-gu, Seoul, Korea

**Keywords:** Knee, High tibial osteotomy, Closing wedge, Navigation, Experience

## Abstract

**Background:**

There is debate regarding the influence of a surgeon’s experience with computer-assisted surgery (CAS) on the postoperative mechanical axis (MA) in CAS-high tibial osteotomy. The purpose of the present study was to compare radiographic results between early and late cohorts of a consecutive series of patients to assess the influence of CAS experience on accuracy and precision of the postoperative MA during CAS lateral closing-wedge high tibial osteotomy (LCWHTO).

**Materials and methods:**

Results from 140 CAS-LCWHTO operations were retrospectively reviewed. The first 70 cases, performed during the learning curve period for CAS between 2005 and 2009, were considered to be the “early cohort.” The subsequent 70 cases, performed with greater CAS experience after the completion of the learning curve between 2009 and 2014, were considered to be the “late cohort.” The target postoperative MA angle was valgus 3°. Pre- and postoperative MA angles were evaluated by navigation and radiographs. The proportion of postoperative MA inliers (≤ target angle ±3°) was investigated radiographically. The correlation between the navigation and radiographic measurements was analyzed.

**Results:**

The average postosteotomy MA angle on navigation was 3.4° in both cohorts. The average postoperative MA angle on radiographs was 1.0° in the early cohort and 2.2° in the late cohort (*P* = 0.003). Radiographically, the proportion of postoperative MA inliers was greater in the late cohort than in the early cohort (early versus late, 71.4% versus 90%; *P* = 0.011). The pre- and postoperative correlation between navigation and radiographic measurements was also stronger in the late cohort (early versus late; preoperative *r* = 0.558 versus 0.663; postoperative *r* = 0.310 versus 0.376).

**Conclusions:**

Greater experience with CAS increased the accuracy and precision of postoperative MA alignment as well as the correlation between navigation and radiographic measurements. Caution should be taken during registration procedures to achieve accurate alignment correction in CAS-LCWHTO.

## Background

The success of high tibial osteotomy (HTO) depends on the accuracy of alignment correction [1]. Under- and over-correction of the mechanical axis (MA) are the main reasons for clinical failure [2]. Several conventional methods to achieve proper postoperative MA are available, including the cable method, use of a grid with lead-impregnated reference lines, or the use of a jig system [3–5]. However, it is difficult to achieve ideal correction consistently using conventional techniques due to occasionally unreliable preoperative planning and static measurement methods [5, 6]. A computer-assisted technique using navigation has been applied recently to allow for intraoperative real-time dynamic measurement of limb alignment. Many studies have reported that navigation increased the correction accuracy in HTO [7, 8].

There is debate regarding the influence of a surgeon’s experience with computer-assisted surgery (CAS) on the postoperative MA in CAS-HTO. Lutzner et al. [9] reported that navigation provides precise information concerning the MA regardless of the surgeon’s experience with CAS. In contrast, Gebhard et al. [10] suggested that the accuracy of the postoperative MA is better when performed by trained CAS surgeons.

Most previous studies evaluating the relationship between navigation and radiographic measurements have reported positive correlations between the two [8, 9, 11–13], although other data are equivocal [14]. Differences between the two measurement techniques can be attributed to errors during manual registration, particularly during HTO with image-free navigation [15].

The purpose of the present study was to compare radiographic results between early and late cohorts of a consecutive series to assess the influence of the surgeon’s experience with CAS on the accuracy and precision of the postoperative MA in CAS lateral-closing wedge HTO (LCWHTO). In addition, this study evaluated the correlation between navigation and radiographic measurements in early and late cohorts. We hypothesized that greater experience with CAS would improve the accuracy and precision of the radiographic results, and lead to a strong correlation between navigation and radiographic measurements.

## Materials and methods

### Patients

Data were obtained from a consecutive series of patients who underwent CAS-LCWHTO between 2005 and 2014. The Vector Vision® computed tomography (CT)-free navigation system (ver. 1.1; BrainLAB, Heimstetten, Germany) was used to measure alignment, and a Miniplate staple (U&I®; Uijungbu-si, South Korea) was used as a fixative. The inclusion criterion for CAS-LCWHTO was medial compartment osteoarthritis (Kellgren-Lawrence grades 3–4) associated with varus deformity. The exclusion criteria were: severe varus deformity > MA angle of 15°; flexion contracture >15°; flexion angle <90°; lateral compartment osteoarthritis (Kellgren-Lawrence grades 3–4); lateral tibial subluxation >10 mm; and diseases other than degenerative osteoarthritis, such as inflammatory or traumatic arthritis. A total of 140 cases of CAS-LCWHTO (130 patients) were included in this study. All operations were performed by a single surgeon using the same technique.

Patients were categorized into two groups considering the learning curve for CAS. It was considered that the CAS-LCWHTOs were performed with greater CAS experience obviously after completion of the learning curve for CAS compared with the cases performed before the learning curve completion. Because the learning curve for CAS-LCWHTO is not well defined, we referred to the previously reported learning curve for CAS in various fields; the learning curves for CAS were completed in 20–70 cases [16, 17]. In the present study, it was determined that 70 cases would be required to complete the learning curve for CAS-LCWHTO. The first 70 cases, performed during the learning curve period for CAS between 2005 and 2009, were considered to be the “early cohort.” The subsequent 70 cases, performed with greater CAS experience after the completion of the learning curve between 2009 and 2014, were considered to be the “late cohort.”

The study was approved by the Institutional Review Board of our institution. Written informed consent was obtained from all patients prior to review.

### Surgical technique and rehabilitation

Identical surgical techniques and registration procedures were used for both the early and late cohorts. CAS-LCWHTO was performed as described previously [8]. The standard registration procedure was conducted according to the requirements of the navigation system used. The target postoperative MA angle was 3°, and the target MA percentage (MA%) was 62% [18].

A similar rehabilitation protocol was used for all patients. Isometric exercises were recommended on the operative day, range-of-motion and straight-leg-raising exercises were started 2 days postoperatively, partial weight bearing was started 3 to 5 days postoperatively, and full weight bearing without crutches was started at 6 to 12 weeks depending on the patient’s condition.

### Radiographic evaluation

Radiographic parameters were measured on preoperative radiographs and on radiographs taken 3 months postoperatively to evaluate the accuracy of surgery. The 3-month follow-up period was selected to address concerns that these parameters might be influenced by rehabilitation and patient compliance with weight bearing.

Radiographic measurements of coronal alignment, including the MA angle and MA%, were obtained from full-length, weight-bearing orthoroentgenograms, which included the hip, knee, and ankle. Lateral radiographs of the knee were obtained and reviewed to assess the tibial posterior slope angle (PSA).

High-quality standardized pre- and postoperative radiographs were obtained for all patients [19]. To ensure the quality of the radiographic evaluation, the radiographic protocol involved standardization of the position of the knee. The orthoroentgenograms were taken with the patient standing with the knee fully extended and the feet slightly internally rotated to ensure forward placement of the patella. For the lateral radiographs, the knee was positioned in the same manner as for the orthoroentgenograms, except the x-ray beam was directed laterally, 90° to the anteroposterior view. The images were transferred digitally to a picture archiving and communication system (PACS). Assessment was performed on a 61-cm monitor (SyncMaster 2494HMN; Samsung, Seoul, South Korea) in portrait mode with PACS software (Infinitt, Seoul, Korea). The minimum angular difference that the software could detect was 0.1° [20].

**Fig. 1:**
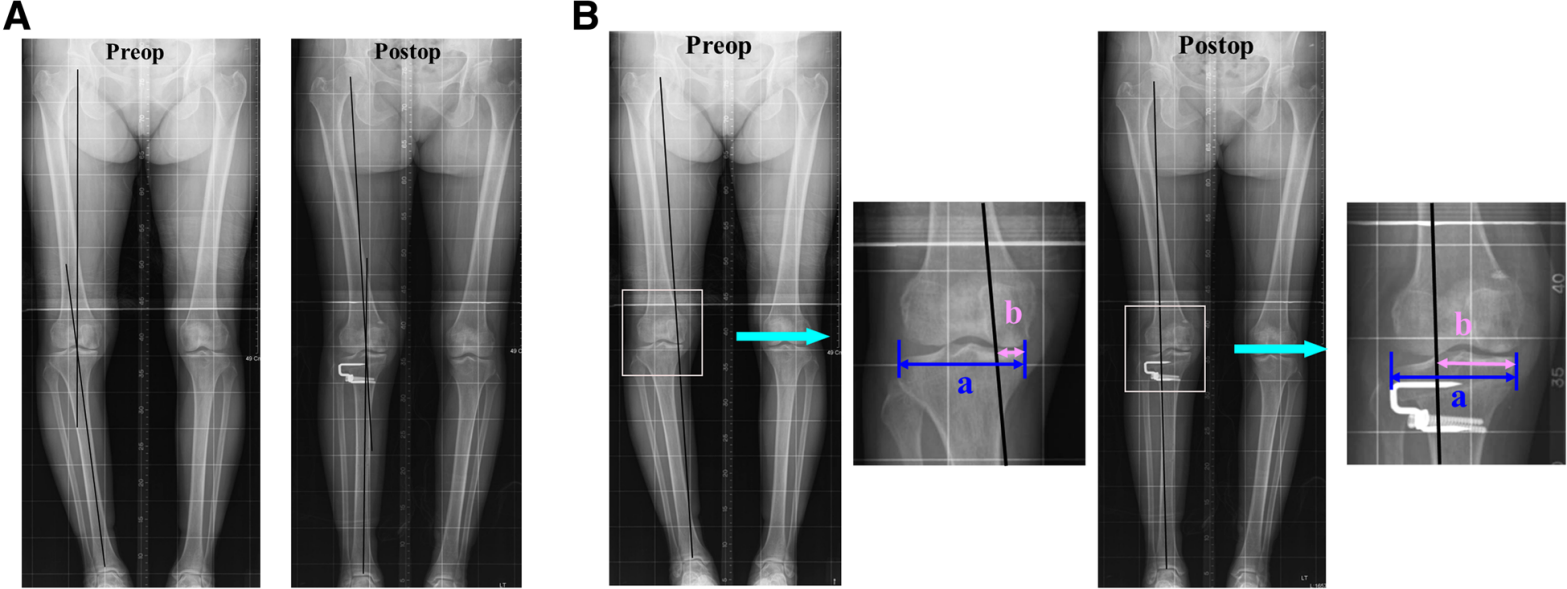
Radiographic measurement of the preoperative (preop) and postoperative (postop) mechanical axis (MA) and the percentage of the mechanical axis (MA%). **a** The MA was defined as the angle between the femoral and tibial mechanical axes on an orthoroentgenogram. **b** The MA% shown on the orthoroentgenogram was evaluated by percentile denotation ([b/a] × 100), where a is the width of the tibia plateau and b is the distance from the medial border of the medial tibial condyle to the point at which the mechanical axis intersects the knee joint line

The MA angle was defined as the angle between the femoral and tibial mechanical axes (Fig. 1). The MA% was defined as the percentage at which the line connecting the centers of the hip and talus bisected the total width of the tibia (Fig. 1b). The PSA was measured with a reference line connecting the center of the medullary canal 10 cm and 20 cm distal to the tibial plateau; it was defined as the angle between the reference line and a line connecting the anterior and posterior borders of the medial tibial plateau.

Postoperative MA inliers were defined as knees with a postoperative MA angle within the target angle (valgus 3°) ± 3°. The inliers for the change in the PSA (postoperative PSA – preoperative PSA) were defined as knees with a change within ±2° (i.e., within the range of clinical significance) [21].

To reduce bias, two independent investigators performed all radiographic measurements. The interobserver reliability of the measurements was assessed using intraclass correlation coefficients; these were >0.8, indicating good reliability. The radiographic measurements that were taken by the investigator with more clinical experience were used in the analyses.

### Measurement on navigation

Under navigation guidance, the MA angle was measured before the osteotomy. The postosteotomy MA angle and MA% values were measured after wedge closing and fixation.

### Complications

Any complications that might affect the radiographic outcomes were recorded.

### Statistical analysis

Patient demographics, including age, sex, body mass index, and operative side, were compared between the early and late cohorts with independent *t* or chi-square tests. Pre- and postoperative MA angles and MA% values, on navigation and radiographs, were compared between the early and late cohorts with independent *t* tests. Likewise, the pre-and postoperative PSA, and the change in the PSA on radiographs, were compared between the two groups with independent *t* tests. The proportion of inliers for the postoperative MA and change in PSA, which were radiographically evaluated, were compared with chi-square tests. The correlations between navigation and radiographic measurements for the pre- and postoperative MA angles were assessed with Pearson correlation analyses. Statistical analyses were performed with SPSS for Windows (ver. 18.0; SPSS Inc., Chicago, IL, USA). *P* values <0.05 were considered statistically significant.

Post hoc power analyses using significance levels set to an alpha of 0.05 were performed to determine whether the sample had sufficient power to detect significant differences. A power >80% was considered sufficient, and all variables that were significantly different met this criterion.

## Results

### Demographics

There were no significant differences between the early and late cohorts in age, sex, body mass index, or operative side (Table 1).

**Table 1:** Demographics of early and late cohorts in computer-assisted lateral closing-wedge high tibial osteotomy

	Early cohort	Late cohort
Operative period	2005–2009	2009–2014
Number of patients	65	65
Number of knees	70	70
Age (years)	59.2 ± 7.7	57.7 ± 5.5
Sex (female/male)	61/4	59/6
Body mass index (kg/m^2^)	25.0 ± 2.6	24.9 ± 2.3
Right/left	41/29	43/27

### Radiographic results

The preoperative MA angle did not differ significantly between the two cohorts (*P* = 0.078; Table 2). The average postoperative MA angle on radiographs was 1.0° valgus in the early cohort and 2.2° valgus in the late cohort (*P* = 0.003). There were no significant differences between the two cohorts in the pre- or postoperative PSA, or the change in the PSA (Table 2).

**Table 2:** Comparison of navigation and radiographic measurements between early and late cohorts of computer-assisted, lateral closing-wedge high tibial osteotomy

		Early cohort	Late cohort	*P* value
Navigation				
Mechanical axis (°)^a^	Preoperative	Varus 8.4 ± 2.7	Varus 9.0 ± 3.2	0.204
	Postoperative	Valgus 3.4 ± 1.4	Valgus 3.4 ± 1.4	0.894
Mechanical axis % (%)^b^	Postoperative	62.1 ± 5.8	61.0 ± 6.5	0.140
Radiograph				
Mechanical axis (°)^a^	Preoperative	Varus 7.3 ± 3.1	Varus 8.3 ± 3.0	0.078
	Postoperative	Valgus 1.0 ± 2.9	Valgus 2.2 ± 1.7	**0.003**
Mechanical axis % (%)^b^	Preoperative	14.8 ± 11.9	10.8 ± 14.3	0.079
	Postoperative	55.5 ± 11.5	60.8 ± 8.3	0.138
Tibial posterior slope angle	Preoperative	10.2 ± 2.1	10.1 ± 2.3	0.764
	Postoperative	8.3 ± 2.2	8.4 ± 2.3	0.694
Change in tibial posterior slope angle		−1.9 ± 0.7	−1.7 ± 1.0	0.084

^a^Mechanical axis, angle between the femoral and tibial mechanical axis; negative values indicate varus angles; ^b^mechanical axis %, percentile denotation ([b/a] × 100) of the point at which the mechanical axis of the lower extremity intersected the line extending from the medial border to the lateral border of the tibial plateau on orthoroentgenogram

Significant values are shown in bold typeface; *P* <  0.05

The proportion of postoperative MA inliers was significantly greater in the late cohort than the early cohort (early versus late, 71.4% versus 90%; *P* = 0.011; Table 3). The proportion of inliers for the change in the PSA did not differ between groups (early versus late, 92.9% versus 97.1%; *P* = 0.061; Table 4).

**Table 3:** Angular distribution of the postoperative mechanical axis between early and late cohorts of computer-assisted lateral closing-wedge high tibial osteotomy

Postoperative mechanical axis (°)	Early cohort	Late cohort
< Varus 2	10	0
Varus 2–0	7	5
0 to Valgus 2	25	26
Valgus 2–4	23	29
Valgus 4–6	2	8
Valgus 6–8	3	2
> Valgus 8	0	0
Total	70	70

**Table 4:** Distribution of change in the tibial posterior slope angle between early and late cohorts of computer-assisted lateral closing-wedge high tibial osteotomy

Change in tibial posterior slope angle (°)	Early cohort	Late cohort
−4 to −2	4	2
−2 to 0	58	61
0–2	7	7
2–4	1	0
Total	70	70

### Measurement on navigation

Under navigation guidance, there was no significant difference between early and late cohorts in the postoperative MA angle or MA% values (Table 2).

### Correlation between navigation and radiographic measurements

**Fig. 2:**
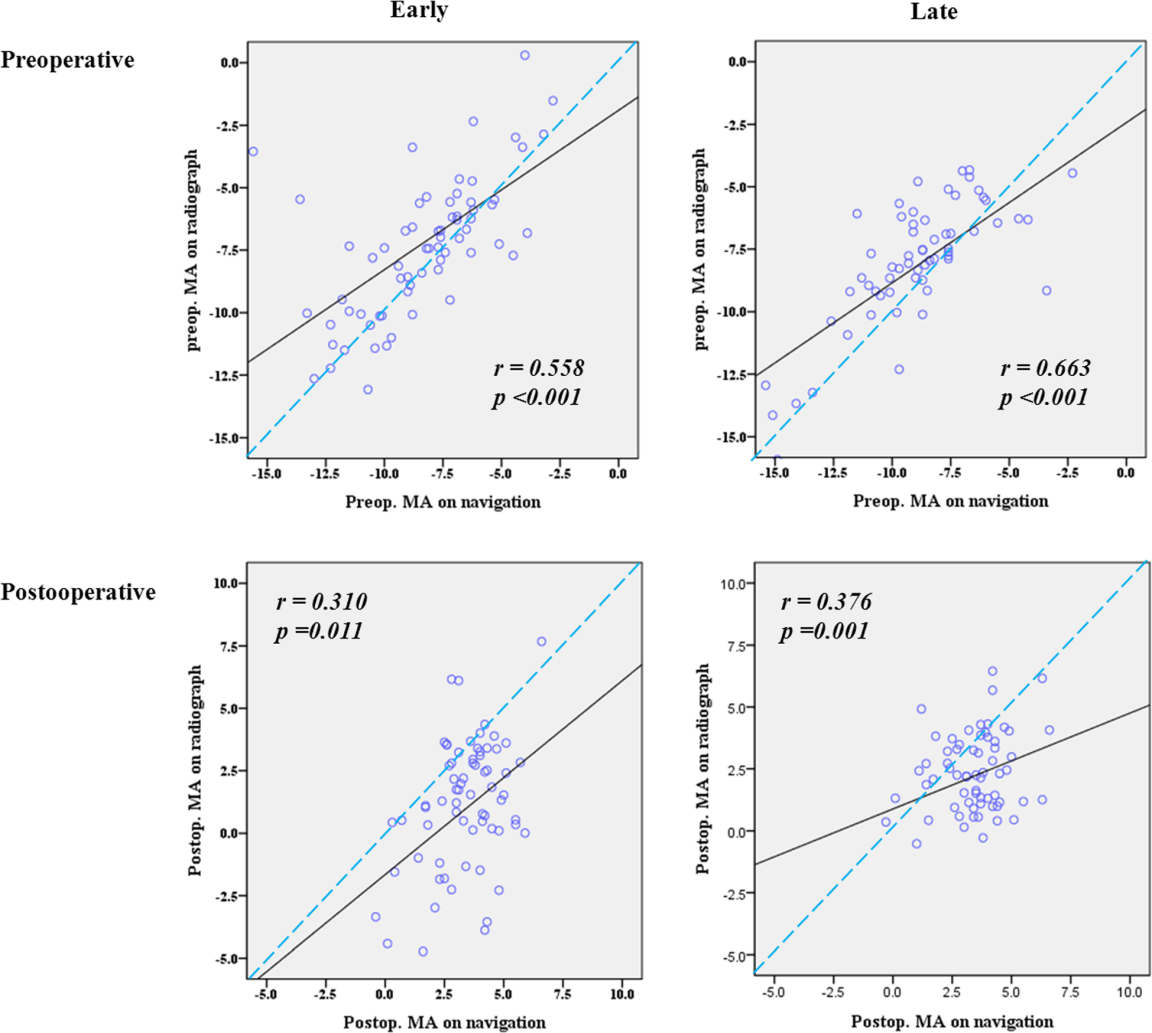
Correlation between navigation and radiographic measurements of the preoperative (preop) and postoperative (postop) mechanical axes (MA). There was a positive correlation between the navigation and radiographic measurements of the pre- and postoperative MA alignment. The intraclass correlation coefficients indicated that the pre- and postoperative correlation between the navigation and radiographic measurements was stronger in the late cohort than the early cohort (preoperative: late cohort, 0.663; early cohort, 0.558; postoperative: late cohort, 0.376; early cohort, 0.310). The reliability of MA alignment on navigation was decreased after osteotomy and wedge closing in both the early and late cohorts, although there was a positive correlation between the results obtained using the two methods

In both cohorts, there were positive correlations between the navigation and radiographic measurements for the pre- and postoperative MA angles (Table 5). However, the pre- and postoperative correlation between navigation and radiographic measurements was stronger in the late cohort (Table 5). The correlation coefficient decreased after osteotomy and wedge closing in both cohorts, although there were still positive correlations between the navigation and radiographic measurements (Table 5; Fig. 2).

**Table 5:** Comparison of the intraclass correlation coefficient between radiographic and navigation measurements in early and late cohorts of computer-assisted lateral closing-wedge high tibial osteotomy

		Early cohort	Late cohort	Total
Preoperative mechanical axis (°)^a^	*r*	0.558	0.663	0.618
	*P*	<0.001	<0.001	<0.001
Postoperative mechanical axis (°)^a^	*r*	0.310	0.376	0.329
	*P*	0.011	0.001	<0.001

^a^Mechanical axis, angle between the femoral and tibial mechanical axes; negative values indicate varus angles

### Complications

No complications, such as infection, delayed union, nonunion, or malunion, occurred.

## Discussion

The most important finding of the present study was that greater experience with CAS was associated with increased accuracy and precision in the postoperative MA in CAS-LCWHTO. In the late cohort, the mean postoperative MA angle on radiographs was significantly closer to the target angle than in the early cohort. Likewise, the proportion of inliers for the postoperative MA was greater in the late cohort.

The reason that the early cohort showed inferior postoperative radiographic results, despite the use of CAS, might be registration error (the errors in registration of the anatomical landmarks) due to the surgeon’s limited experience with CAS. Although navigation may improve coronal alignment by using real-time intraoperative measurements, there is still the potential for inaccuracy. This may be partly attributable to errors during manual registration of the anatomical landmark, which is performed to establish knee and ankle centers for defining the mechanical axis and the osteotomy level of the navigation system. The accuracy of manual registration depends on the surgeon’s experience with CAS; registration errors have been shown to occur if surgeons do not have sufficient CAS experience [22]. Because the use of a computer cannot compensate for failure to accurately localize landmarks, such an error will inevitably lead to a different postoperative MA alignment than planned preoperatively. Yau et al. [22] reported that registration errors during acquisition of visually selected landmarks induce projected errors of the femoral and tibial mechanical axes on the navigation system.

To demonstrate that the above hypothesis explains our findings, we also investigated the correlation between navigation and radiographic measurements to assess the reliability of registration procedures in early and late cohorts. The pre- and postoperative correlation was stronger in the late cohort when the surgeon had greater CAS experience. Although several studies have reported a correlation between navigation and radiographic measurements of MA [6, 23], our study is the first to investigate the influence of surgeon experience on the correlation between navigation and radiographic measurements.

Therefore, surgeons should be aware that mistakes occurring during the registration procedure can result in significant errors in postoperative MA in CAS-HTO. Caution should be taken during registration procedures to avoid errors and achieve accurate alignment correction [9].

Notably, the strong preoperative correlation between navigation and radiographic measurements decreased postoperatively in the present study, which is consistent with previous findings [12, 14]. This might be explained by the fact that fibular management and wedge closing during LCWHTO can alter soft tissue tension and, finally, postoperative alignment on weight-bearing radiographs. Fibular management might affect the integrity of the lateral collateral ligament and posterolateral structures of the knee [20]. The lateral wedge closing might decrease tension of the medial collateral ligament secondary to functional laxity away from the center of rotation [24]. Surgeons will be able to improve the reliability of navigation measurements for postoperative MA alignment by considering alterations in soft tissue tension after wedge closing during CAS-LCWHTO.

The present study had several limitations. First, it was a retrospective study with a relatively small cohort. A prospective study with a larger cohort will be required to achieve more robust conclusions. Second, there could be limitations to the accuracy of the radiographic measurements. Small changes in the projection angle and rotation, or flexion of the knee, could have affected the radiographic measurements. Although CT can accurately measure limb alignment, radiation exposure limits the use of CT. Instead, we attempted to acquire consistent films in a standardized knee position, and we confirmed the intra- and interobserver reliability of all measurements. Third, we did not investigate other variables related to the proficiency of registration, such as registration time. Investigating this variable would have better validated our hypothesis that the differences between the early and late cohorts were due to registration errors. Last, we did not perform a clinical evaluation. There is no direct evidence that CAS-HTO leads to superior long-term outcomes, although it has been shown to improve the accuracy of postoperative limb alignment. Assuming that proper postoperative alignment results in clinical satisfaction, we focused on the accuracy and precision of the radiographic measurements of MA alignment.

## Conclusion

Greater experience with CAS increased the accuracy and precision of postoperative MA alignment, as well as the correlation between navigation and radiographic measurements. Caution should be taken during registration procedures to achieve accurate alignment correction in CAS-LCWHTO.

## Data Availability

The datasets generated and/or analyzed during the current study are not publicly available, but they are available from the corresponding author on reasonable request.
